# A Comparative Study of Circadian Rhythmicity and Photoperiodism in Closely Related Species of Blow Flies: External Coincidence, Maternal Induction, and Diapause at Northern Latitudes

**DOI:** 10.1177/07487304211054419

**Published:** 2021-11-05

**Authors:** David Saunders

**Affiliations:** The University of Edinburgh (Professor Emeritus), Edinburgh, UK

**Keywords:** blow flies, Bünning’s hypothesis, photoperiod, diapause, ‘hands’ of the clock, external coincidence, latitude

## Abstract

This review compares adult locomotor activity rhythms and photoperiodic induction of diapause in 3 common species of blow fly, *Calliphora vicina, Lucilia sericata*, and *Protophormia terraenovae.* Activity rhythms were broadly similar in all 3 species, although *P. terraenovae* is much less sensitive to constant light inducing arrhythmicity. Photoperiodic induction of diapause, on the other hand, varies more widely between species. *C. vicina* and *L. sericata* overwinter in a larval diapause induced by autumnal short days (long nights) acting both maternally and directly upon the larvae. *P. terraenovae*, on the other hand, shows an adult (reproductive) diapause induced by short daylength and low temperature experienced by the larvae. In the Nanda-Hamner protocol, *C. vicina* shows 3 clear peaks of high diapause incidence in cycle lengths close to 24, 48, and 72 h, without dampening and therefore suggesting a photoperiodic mechanism based on a self-sustained circadian oscillator acting in a clock of the external coincidence type. Entrainment of the locomotor activity rhythm to extended Nanda-Hamner photocycles, as well as to LD cycles close to the limits of the primary range of entrainment, demonstrates that overt circadian rhythmicity may act as ‘hands’ of the otherwise covert photoperiodic system, as suggested by Bünning, nearly 8 decades ago. In 24 h LD cycles, both locomotor activity rhythms and the photoperiodic oscillator are set to constant phase (CT 12) at light-off, so that the photoperiodic clock measures changes in nightlength by the coincidence (or not) of dawn light with a ‘photoinducible phase’ late in the subjective night (at about CT 21.5 h) as photoperiod changes with the seasons. Apparent differences between quantitative and qualitative photoperiodic responses are discussed.

Bünning (1936, 1960), working with plant photoperiodism and later with insects (Bünning and Joerrens, 1960), proposed that photoperiodic time measurement was a function of the circadian system. He also suggested that visible manifestations of the circadian system–such as overt rhythms–could be used as ‘hands of the photoperiodic clock’ to facilitate investigation of the otherwise ‘hidden’ system. This approach, which has already been used in analyses of diapause induction in several insect species (Saunders, 2020, 2021) is now extended to *Calliphora vicina.*

Larval development of blow flies (Diptera: Calliphoridae) occurs in vertebrate carcases and contributes to the elimination of carrion from the ecosystem. These flies also have a more direct economic importance, laying eggs and developing as larvae in living tissue (myiasis) of domestic animals, particularly sheep (Hall and Wall, 1995). In human corpses the time course of larval infestations is also used, in forensic entomology, to determine the date and time of death (Amendt et al., 2021), and in human medicine they may assist wound healing by their use in ‘maggot therapy’ (Harvey et al., 2021). The common, urban ‘bluebottle’ *Calliphora vicina* and the ‘greenbottle’ fly *Lucilia sericata* investigated in this paper are widespread in Europe and frequently involved in cases of ‘sheep strike’. The third species, the ‘blackbottle’ fly *Protophormia terraenovae*, also causes myiasis, but has a more northerly distribution, occurring as far north as Baffin Island and Spitzbergen. All 3 species are considered here because aspects of their circadian biology and photoperiodism have been subjects of intensive study in the laboratory, in addition to their roles in veterinary and forensic medicine.

In this review circadian rhythmicity and the photoperiodic induction of diapause in these related blow flies are compared. In the first two sections circadian rhythmicity and photoperiodic induction are reviewed separately because these topics have been considered in the past as separate phenomena. In the third section, events occurring from photoreception, through central aspects of time measurement to their endocrine or neural outputs are compared more directly to present similarities and differences between the species. Particular attention is given to the maternal induction of diapause, latitudinal clines, the nature of time measurement and the transmission of seasonal information from photoreception to output mechanisms.

## Circadian Regulation of Locomotor Activity

**Figure 1.**
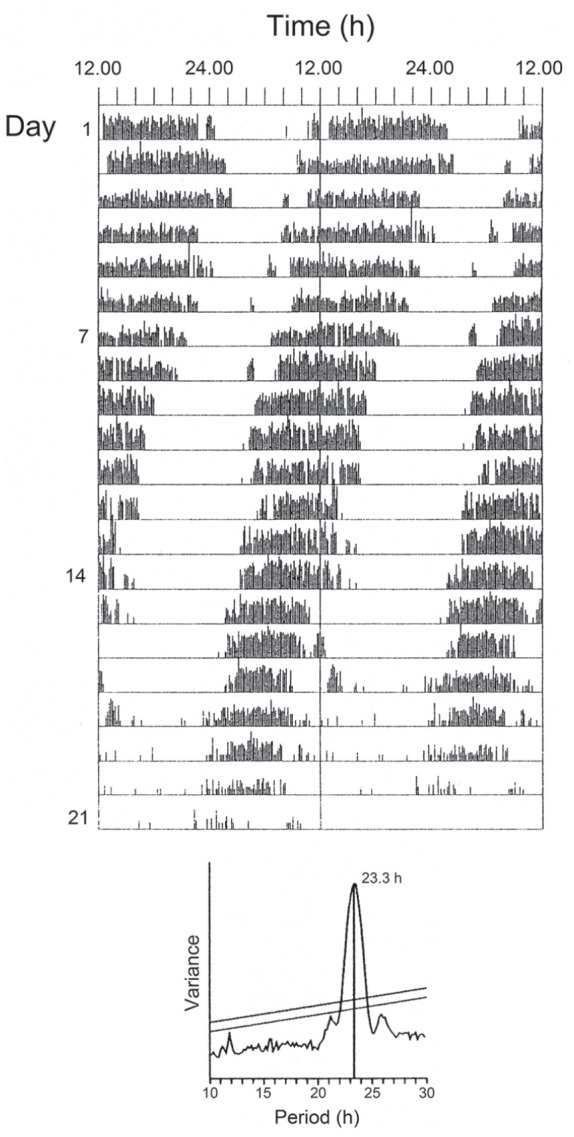
*Calliphora vicina.* Locomotor activity rhythm of a female fly at 20 °C free-running in darkness (τ_DD_) shown as a ‘double-plotted’ actogram. Periodogram analysis (lower panel) shows τ_DD_ less than 24 h.

Locomotor activity rhythms are well presented in all of the blow flies considered in this review. However, the most complete of these studies is that for *Calliphora vicina* using a strain of flies isolated in mid-Scotland (55 ^o^ N). These studies established the canonical features of circadian rhythmicity, including persistence (free-running) of the rhythm in constant darkness (DD), temperature compensation of its period and its entrainment by environmental light and temperature cycles (Kenny and Saunders, 1991; Hong and Saunders, 1994; Saunders and Hong, 2000). Figure 1 shows a typical activity record for *C. vicina* with rhythmicity largely self-sustained and free-running in continuous darkness (DD) for at least 2 weeks, with a period (τ) less than 24 h.

**Figure 2.**
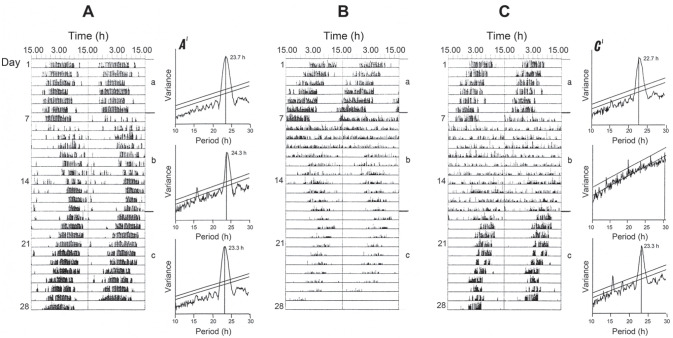
*Calliphora vicina*. Effect of light intensity on circadian period in extended periods of light, female flies at 20 °C, held for the first 7 days in DD, then for 11 days in LL at various light intensities, and finally in DD. (a)—fly in ‘dim’ light (0.018 Wm^-2^), (b)—in ‘intermediate’ light (0.024 Wm^-2^), (c)—in ‘bright’ light (0.033 Wm^-2^). A’ and C’ show periodogram analyses for the appropriate sections of actograms A and C. Arrows show the times of light on and light off. From Hong and Saunders (1994). Abbreviation: DD = constant darkness.

The rhythm of activity in *C. vicina* may also persist in constant light (LL) but only under low light intensity (Hong and Saunders, 1994). For example, Figure 2a shows the transfer of a fly from DD into constant dim light at 0.018 Wm^-2^ (about 12.3 lux) resulting in a lengthening of τ from 23.7 h to 24.3 h (and back to τ 23.3 h after return to DD), whereas a similar transfer from DD into brighter light at 0.033 Wm^-2^ (about 22.6 lux) (Figure 2c) induced behavioural arrhythmicity. At an intermediate light intensity (0.024 Wm^-2^ or about 16.4 lux; Figure 2b) activity was initially arrhythmic but then after about 5 days became rhythmic with a period longer than 24 h. This suggests that the fly initially perceived the light as ‘bright’, but later as ‘dim’, as the photoreceptors accommodated to the light. The threshold light intensity for the change from τ_LL_ > 24 h to arrhythmia is therefore about 0.024 Wm^-2^.

**Figure 3.**
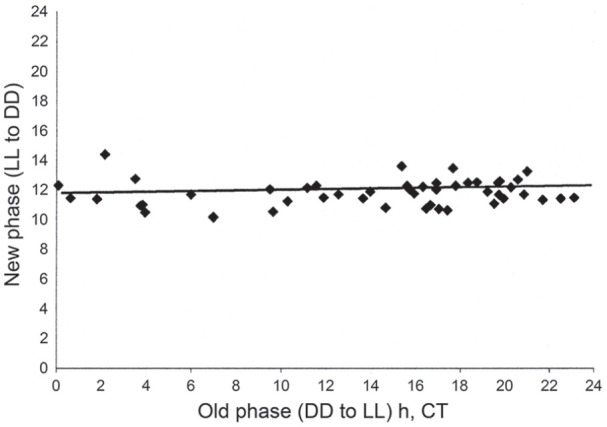
*Calliphora vicina.* Phase transition curve for flies exposed to an extended period of ‘bright’ light (0.035 Wm^-2^) starting at all circadian times (old phase), showing that the activity rhythm is phase set to a near constant (new) phase close to Circadian Time (CT 12 h): regression y = 11.521 + 0.021 x. From Saunders and Cymborowski (2008). Abbreviations: LL = constant light; DD = constant darkness.

Working with the rhythm of adult eclosion in *Drosophila pseudoobscura*, Pittendrigh (1966) showed that emergence of adult flies in a mixed-age population also became arrhythmic in LL, but a rhythm became re-apparent after transfer to DD, starting again at a particular point denoted as Circadian Time, CT 12 h (the start of the ‘night’ phase of the endogenous rhythm) upon transfer to darkness. This same phase relationship at the point of transfer to DD was also observed at the end of a light period longer than about 12 h, so that the oscillation always recommenced from CT 12 at the onset of the night following a lengthy light phase. A similar result (Figure 3) has since been observed with the locomotor activity rhythm of the blow fly *C. vicina* (Saunders and Cymborowski, 2008), in which periods of constant bright light (48 lux) of up to 7 days duration, commencing at any circadian phase, set the oscillation close to CT 12 at light-off. This observation has crucial importance to the understanding of photoperiodic time measurement in this and other species (see below) and locomotor activity rhythms have proved to be useful ‘hands’ of the covert photoperiodic clock (Kenny and Saunders, 1991).

Comparable locomotor activity records are not currently available for *Lucilia sericata*, but studies using the Australian sheep blow fly *L. cuprina* were described by Smith (1983). This study used male flies usually in groups of 10 in cages measuring 20 cm x 20 cm x 20 cm. Activity measured by their spontaneous flight in light-dark cycles showed that *L. cuprina* males were day-active, but with considerable activity during the dark phase. However, flies maintained individually showed activity records very similar to those described above for *C. vicina*: almost all activity occurred in the photophase and a transfer from LD 12:12 h into darkness revealed a free-running (and therefore endogenous) rhythmicity with τ values between 21.75 and 22.75 h. Rhythmicity also persisted after transfer of flies from LD to continuous dim light (dim LL) of less than 1 lux, but brighter light resulted in arrhythmic behaviour. Similar results were later obtained by Warman and Lewis (2001) for females of *L. cuprina* free-running in DD with τ of about 22 h, but in dim LL (0.001 W m^-2^) with an increase to 24.2 h. Transfer to a brighter light (50 W m^-2^) also caused activity to become arrhythmic, as in *C. vicina*.

Locomotor activity rhythms of *Protophormia terraenovae* were first recorded by Aschoff and von Saint Paul (1982) using small (9 cm diameter) Perspex running wheels. Unlike *C. vicina* and *L. cuprina* no activity was detected in DD, or in dim light below about 1 lux. This result, however, might be a consequence of the type of activity recorder used since later studies with *P. terraenovae* using infra-red detectors showed free-running rhythms in DD with a period of about 25 h (Hamasaka et al., 2001). Furthermore, in brighter LL (120 lux)–an intensity known to induce arrhythmicity in *C. vicina* and *L. cuprina*—these authors showed that most *P. terraenovae* continued to present persistent free-running rhythms of activity with a long period. In a later paper, Hamasaka et al. (2011) showed that in both wild-type and white-eye mutant *P. terraenovae* lacking screening pigment, more than half the cases were still rhythmic in LL at 500 lux. *P. terraenovae*, therefore, has a much higher threshold than either *C. vicina* or *L. cuprina* for the intensity of light causing arrhythmia.

## Photoperiodic Induction of Diapause

In the bluebottle *Calliphora vicina*, maternal exposure to short-day photoperiod is the principal environmental factor inducing larval diapause, provided that the resulting larvae are also exposed to temperatures below about 15 °C (Vinogradova and Zinovjeva, 1972; Saunders, 1987). Direct exposure of the larvae to short photoperiod is also a diapause-inducing factor (Vinogradova, 1974; Vaz Nunes and Saunders, 1989); photoperiodic induction is also possible in embryos once the CNS has developed. Imaginal diapause has also been reported in this species (Vinogradova, 1991); *C. vicina*, therefore, is unusual for insects in having more than one potential diapausing stage and photoperiodic sensitivity in several stages of development.

**Figure 4.**
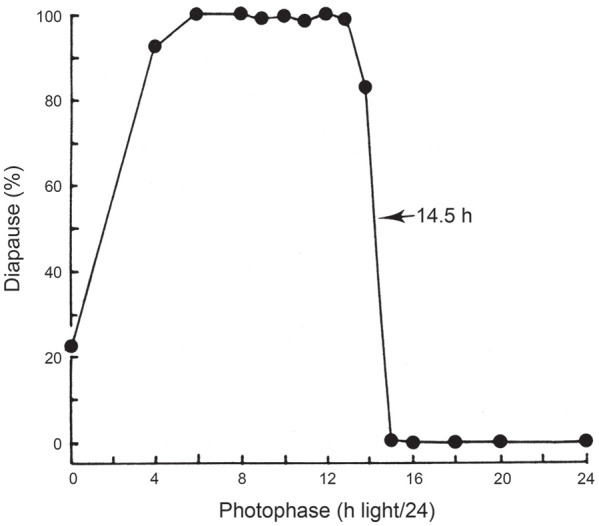
*Calliphora vicina.* Induction of larval diapause by maternally operating photoperiods at 20 °C; larvae reared in constant darkness at 11 °C. Critical daylength (50 % diapause) is at 14.5/24 h. Various sources.

Figure 4 shows a photoperiodic response curve (PPRC) for the strain of *C. vicina* isolated in mid-Scotland (55 ^o^N) (Saunders, 1987) in which adult flies were exposed to various photoperiods at 20 °C and the resulting larvae reared at 11°C in darkness. This PPRC is of the typical ‘long-day’ type, showing continuous, nondiapause development under long summer days, but larvae entering diapause when autumnal days shorten below a well-marked critical daylength (CDL), in this case a CDL of about 14.5 h/24. The abrupt change in diapause incidence at the critical point is diagnostic for a photoperiodic ‘clock’ measuring either the length of the day (or the night) and is a product of natural selection providing a meaningful switch-point between the summer and autumnal developmental pathways appropriate for this particular strain of fly (i.e. one isolated at a latitude of 55 ^o^N). Such a CDL is not uniquely characteristic of a particular species, however; CDLs differ in strains from different latitudes, or at different temperatures. For example, temperature effects are illustrated by the CDL for the Scottish strain at about 14.5 h/24 at 20 °C (Saunders, 1987) but shortening to 13.25 h/24 at 23.5 °C (Vaz Nunes et al., 1990); at 26 °C diapause incidence was low under all photoperiods and a CDL was not apparent.

**Figure 5.**
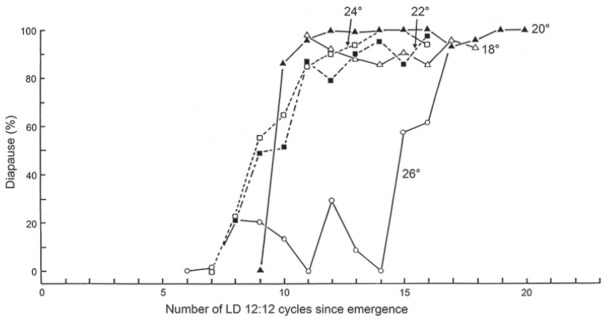
*Calliphora vicina*: the ‘photoperiodic counter’ mechanism. Incidence of larval diapause from egg batches deposited on successive days of adult life and at a range of temperatures (18 to 26 °C) and short daylength (LD 12: 12 h). Apart from the highest temperature, all cultures require about 9 to 10 short-day cycles to ‘switch’ to the production of diapausing offspring. Larvae were reared in darkness at 11 °C. From Saunders (1987).Abbreviations: LD = light:dark.

The incidence of larval diapause developing from egg batches laid on successive days by flies maintained under short days (LD 12:12 h) and at a range of temperatures is compared in Figure 5. These data show that, regardless of temperature between 18 and 24 °C, the incidence of diapausing progeny of *C. vicina* increases with the number of inductive short-day cycles experienced, in an almost temperature-compensated fashion, with about 9 to 10 short-day cycles needed to effect the switch to diapause throughout this temperature range. Only at the highest temperature (26 °C) is this relationship disturbed. Induction of diapause in *C. vicina*, therefore, involves 2 processes that operate concurrently: (1) measurement of daylength (or nightlength) by the photoperiodic clock and (2) the accumulation of this information by a temperature-compensated ‘counter’ mechanism, similar to that previously described for the parasitic wasp *Nasonia vitripennis* (Saunders, 1966) and its flesh fly host, *Sarcophaga argyrostoma* (Saunders, 1971, 1981).

Seminal work by Danilevskii (1965) on Russian populations of the knot grass moth *Acronycta rumicis* showed that CDL varied with latitude, ranging from 14.5 h/24 in the south (Sukhumi, on the Black Sea coast, 43 ^o^N) to about 19.5 h/24 in the north (St Petersburg, 60 ^o^N), lengthening by about 1.5 h with every 5 ^o^ N of latitude. The selective advantage of such a genetic cline suggested that insects at higher latitude compensate for the longer summer daylength but the earlier onset of winter, by having a longer CDL and therefore being able to enter diapause before the first frosts. Similar examples of such clines are now recognised in many species, notable examples being the mosquito *Wyeomyia smithii* (Bradshaw, 1976) and the fruit fly *Drosophila littoralis* (Lankinen, 1986).

**Figure 6.**
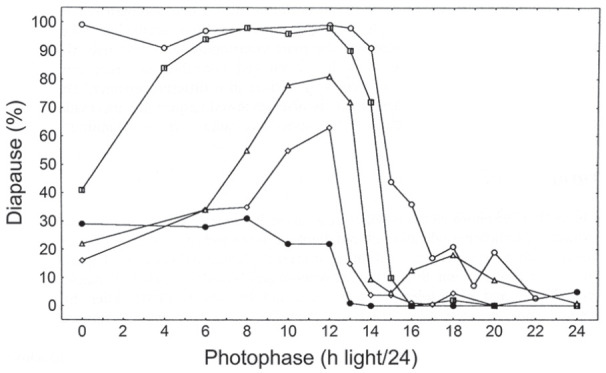
*Calliphora vicina.* Photoperiodic response curves (PPRCs) for flies originating from 5 localities (right to left: Nallikari, Finland, 65 ^o^N, Edinburgh, Scotland, 55 ^o^N, Barga, Italy, 44 ^o^N, Silwood Park, England, 51 ^o^N, Chapel Hill, North Carolina, 36 ^o^N. All PPRCs were conducted at 20 °C. From Saunders (2001).

Figure 6 also shows such a cline for *C. vicina*, using populations originating from northern Finland (Nallikari, 65 ^o^N), central Scotland (Edinburgh, 55 ^o^N), southern England (Silwood Park, 51^o^N) and Italy (Barga, 44 ^o^N); a fifth strain from Chapel Hill, North Carolina (36 ^o^N) is also included in this series which–although not part of the same genetic cline–is an example of a more southerly population (Saunders, 2001). Critical day lengths were found to be longest for Nallikari (15 h/24) and shortest for Silwood Park (12.5 h/24). That for the higher altitude strain from Barga was about 13.5 h/24 (longer than that from Silwood Park) and that from Chapel Hill showed only a weak diapause-inducing response at photoperiods shorter than 12 h. McWatters and Saunders (1998) later showed that the CDL for the strain from southern England (51^o^N) was 14.5 h/24 at 15 °C but shortened to 12.5 h/24 at 20 °C, whereas that for a northern strain from Finland (65 ^o^N) was 16.5 h/24 at 15°C shortening to 15 h/24 at 20 °C.

Apart from critical day length, several other aspects of the photoperiodic response in *C. vicina* show latitudinal variation. These include the number of generations per year (voltinism), with a decrease from a multivoltine life cycle in more southerly localities towards a univoltine life cycle in the north (Vinogradova, 1986), in the ‘switch point’ to diapause production provided by the photoperiodic counter, and in diapause duration (diapause ‘depth’ or ‘intensity’) and in cold tolerance. The intensity or duration of larval diapause in *C. vicina*, for example, is known to be greater among the progeny of females exposed to short days at either a lower temperature or to a greater number of short-day cycles experienced (Saunders et al., 1986; Saunders, 1987). Diapause is also more intense or longer lasting in strains from more northerly locations (McWatters and Saunders, 1996, 1998); those from the Nallikari strain (65 ^o^ N), persisting in diapause for more than 70 days in darkness at 11 to 12 °C, whereas diapause duration for the Barga strain (44 ^o^ N) under the same conditions was only about 30 days.

Reciprocal crosses between parental flies from Nallikari (65 ^o^ N) and the southern strain from Silwood Park (51 ^o^ N) were performed to determine the contributions made by maternal, paternal and latitude-related genes (McWatters and Saunders, 1996). To do this a series of crosses were conducted under LD 15.5:8.5 h at 15 °C, conditions interpreted by the northern strain (N) as diapause-inducing, whereas the southern strain (S) produced nondiapausing progeny. Hybrid crosses between the 2 strains produced a high proportion of diapause larvae (about 90%) when the mother was of the N strain, but a very low incidence of diapause when the female parent was of the S strain; diapause incidence among the larvae, therefore, was determined to a large extent by the adult female, the males being unable to influence diapause incidence among their offspring. However, while diapause incidence was controlled by the female parent, diapause intensity in the N x S hybrids was intermediate between the 2 parental strains indicating that diapause duration is a larval phenomenon influenced by genes from both parents. A later investigation (McWatters and Saunders, 1997) using crosses and backcrosses between the northern and southern populations revealed a maternal grandmother effect with the genes controlling diapause incidence having a greater effect when inherited down the maternal than the paternal line. For diapause intensity, on the other hand, males and females make equal contributions, indicating that intensity is, in contrast to diapause incidence, a purely larval trait.

In *C. vicina* there is also evidence that diapausing larvae are more cold tolerant than nondiapausing larvae (Saunders and Hayward, 1998), and those from more northerly latitudes show a greater degree of resistance to cold than diapausing larvae from localities further south. For example, survival to eclosion, after exposure to temperatures down to −8 °C, was greater for the larval progeny of short-day exposed females from the 2 northern strains (Nallikari, 65 ^o^ N, and Edinburgh, 55 ^o^ N) than those from the southern strain (Barga, 44 ^o^ N). Diapausing larvae from the Scottish strain, however, showed greater cold hardiness than Nallikari, possibly because the longer-lasting snow cover in northern Finland provided increased insulation of the sub-soil overwintering sites. Using a strain of *C. vicina* from the Birmingham area (central England, 52 ^o^ N), Coleman (2014) later employed differential selection for larval diapause incidence over 7 generations of flies raised under short days (LD 12:12 h) at either 15 °C (diapause inducing) or at 20 °C (nondiapause inducing). In the parental generation diapause incidence was between 55% and 65%. In the high diapause line the incidence of diapause became 81% in the F2 generation and 100% by the F5; in the low diapause line diapause fell to less than 20% after 3 or 4 generations.

Experiments on larval diapause induction in *L. sericata* facilitate comparison with those in *C. vicina.* Working with a strain of *L. sericata* from Japan (Osaka, 35 ^o^ N), Tachibana and Numata (2004a, 2004b) confirmed Cragg and Cole’s (1952) earlier conclusion that diapause was influenced by environmental conditions affecting both the larvae and the parental, presumably maternal, stages. They showed that most larvae produced by parents maintained under natural long-day conditions (LD 16:8 h at 25 °C) failed to enter diapause, whereas most larvae produced by the parents under short days (LD 12:12 h at 20 °C) did so. These investigations, however, were confined to just 2 maternal photoperiods and therefore insufficient to establish a complete photoperiodic response curve with an abrupt CDL (as in *C. vicina*, Figure 4).

Unlike *C. vicina* and *L. sericata*, the black blow fly *Protophormia terraenovae* overwinters as an adult, in a reproductive diapause characterised by delayed yolk deposition in the ovaries and fat body hypertrophy. *P. terraenovae* is the dominant blow fly species at more northern latitudes up to the Arctic and Subarctic. Vinogradova (1986) studied the incidence of ovarian diapause in strains of *P. terraenovae* isolated in the former Soviet Union collected at latitudes up to 67 ^o^ N. At the northern end of this distribution the seasonal response approached univoltinism with all flies in each generation entering diapause, even at 25 °C, and under all photoperiods between LD 12:12 h and LD 18:6 h. Further south, diapause occurred in 100% of flies under LD 12:12 h but was reduced to about 40% under LD 20:4 h indicating some sensitivity to long days and partial bivoltine development. Low temperature was found to be a major environmental factor inducing diapause.

Numata and Shiga (1995) studied the induction of ovarian diapause in *P. terraenovae* isolated in Japan (43 ^o^ N), near the southernmost part of its distribution. When the maternal generation was exposed to 4 photoperiods between LD 12:12 h and LD 18:6 h, diapause incidence was found to decline steadily through this range, but in a near linear fashion without a well-defined CDL. In a second experiment, eggs produced by flies raised under LD 18:6 h at 25 °C were exposed, as larvae, to photoperiods of either LD 12:12 h or LD 18:6 h at a range of temperatures between 17.5 and 30 °C. Results showed a marked effect of temperature on adult diapause induction together with a reduced incidence of diapause at LD 18:6 h at all temperatures. These results indicated a substantial effect of temperature on the induction of diapause in *P. terraenovae*, but also the inductive effect of light, even if characteristics of a typical photoperiodic clock were inapparent.

## Components of the Circadian and Photoperiodic Systems

Circadian activity rhythms and photoperiodic induction of diapause resemble each other, showing similar linked series of events from photoreception, through a biological clock to an output controlling either daily activity cycles or a seasonal switch to diapause. This section of the review compares these components in *C. vicina, L. sericata*, and *P. terraenovae.*

### Photoreceptors and Photoreception

**Figure 7.**
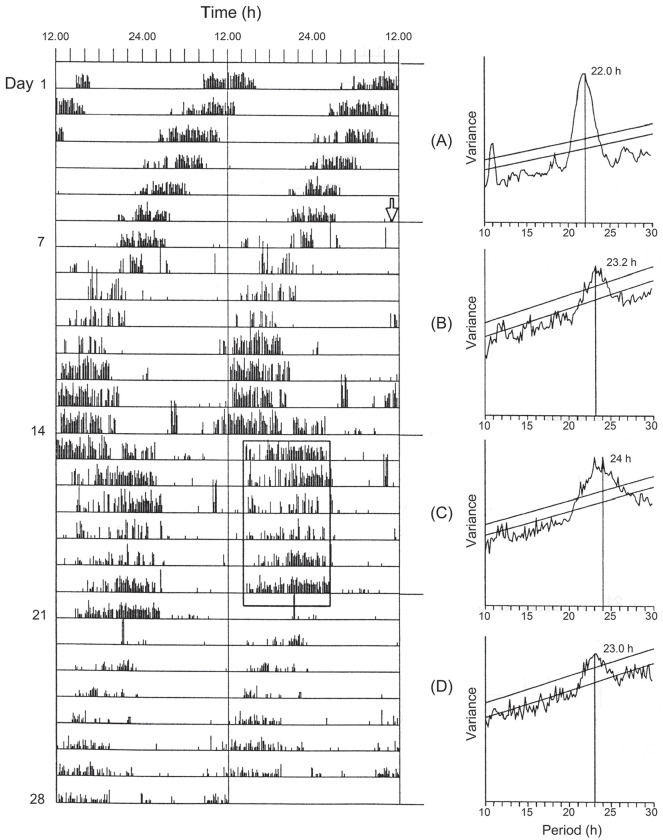
*Calliphora vicina.* Complete removal of the optic lobes (arrow on day 7) leaves free-running locomotor activity rhythm and its entrainment by light cycle (section c) intact, suggesting brain-centred clock location. Right-hand panels show periodogram analyses for the appropriate sections a to d. From Cymborowski et al. (1994).

**Figure 8.**
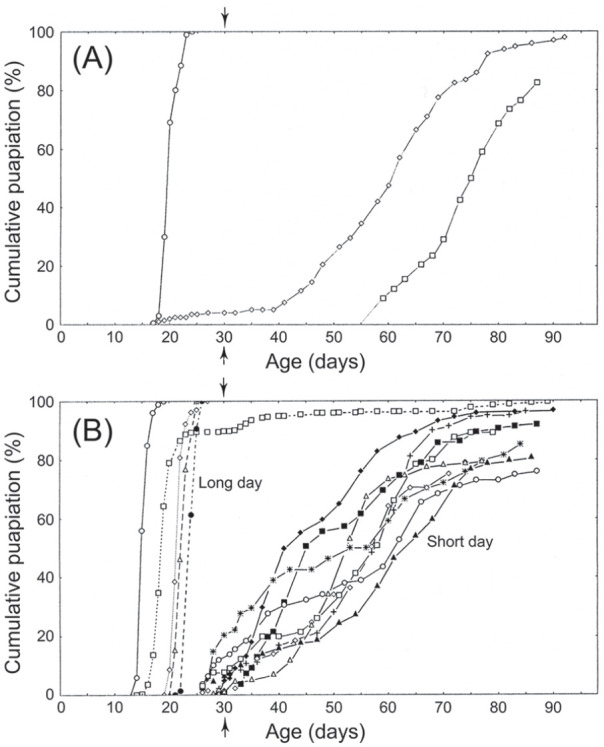
*Calliphora vicina.* Optic lobe removal in adult flies also fails to interrupt photoperiodic regulation of larval diapause, thereby also indicating brain centred photoreception. a—cumulative pupariation curves for larval progeny of unoperated (control) females exposed to either long days (LD 18: 6 h, 20 °C; at left) or to short days (LD 12:12 h, 20 °C; at right). b—ditto for lobectomised females (5 cultures for long-day group; 8 cultures for short-day group). All larval cultures were raised in darkness at 11 to 13 °C, those larvae failing to pupariate by day 30 post eclosion (vertical arrow) were considered to be in diapause. From Saunders and Cymborowski (1996). Abbreviations: LD = light:dark.

In *Drosophila melanogaster*, candidate photoreceptors for the entrainment of circadian locomotor rhythms include compound eyes and extra-optic photoreceptors in the brain (Helfrich-Förster et al., 2001). In blow flies both types of photoreceptor have been investigated. Bilateral optic tract section, or complete bilateral lobectomy in *C. vicina* isolated the compound eyes from the brain but left circadian rhythms of locomotor activity and their entrainment to light cycles intact (Figure 7) (Cymborowski et al., 1994). This suggests that extra-optic photoreceptors operate in the bluebottle, most probably in the central brain, a situation reminiscent of that in some other flies (Helfrich et al., 1985; Kasai and Chiba, 1987). In addition, *C. vicina* females surgically deprived of their optic lobes were shown to retain their ability to distinguish diapause-inducing short days from diapause-averting long days (Figure 8) (Saunders and Cymborowski, 1996), suggesting brain photoreception for both circadian entrainment and photoperiodic induction. Possible photoreceptive inputs to the brain were revealed by the injection of S-antigen (arrestin) antibody into the brain which caused some flies to fail in their entrainment to the LD cycle and to free-run as though they were in DD or in LL of lower intensity (Cymborowski et al., 1996).

Although the compound eyes are not essential for entrainment of the locomotor activity rhythm in *C. vicina* they play an important role in *P. terraenovae* (Hamasaka et al., 2001). When the eyes of this species were covered with silver paint and a black synthetic resin, the locomotor activity rhythm free-ran in LD as though the flies were in DD, whether the photophase was either of dim light (0.5 lux, or 1.4 x 10^-3^ W^-2^) or of much higher intensity (500 lux, or 1.4 W^-2^). Removal of the ocelli had no effect. It was concluded that light passed mainly through the compound eyes from retinal receptors at low light intensities but extraretinal pathways for rhythm entrainment may also occur, perhaps under higher intensity light. Some wild-type and white-eyed mutant flies showed clear rhythmicity under LL of bright light showing that, unlike *C. vicina, P. terraenovae* has a high threshold for arrhythmia (Hamasaka et al., 2011).

Photic regulation of adult (reproductive) diapause in *P. terraenovae* was also found to involve the compound eyes (Shiga and Numata, 1997). Using silver paint to cover the eyes caused diapause incidence to increase under long days and LL as if the flies were in DD, and after bilateral eye ablation all flies developed their ovaries under both long and short days. These experiments leave little doubt that the compound eyes act as photoreceptors for both circadian rhythmicity and diapause regulation, and that there is a significant difference in this respect between *P. terraenovae* and *C. vicina.*

### The Circadian Basis of Photoperiodic Time Measurement

The nature of the insect photoperiodic clock, whether it is a function of the circadian system, a non-repetitive ‘hourglass’, or some other device, has been the subject of intensive enquiry for years (see Vaz Nunes and Saunders, 1999; Saunders, 2020, 2021; Goto, 2013 for recent reviews) and will not be discussed in detail here. Suffice it to say that evidence for circadian rhythmicity playing a causal role in photoperiodic time measurement (PPTM) is strong in some species, but in others less so, suggesting a variety of mechanisms or perhaps a common mechanism with disparate properties. The most frequently suggested models are the hourglass-like timer of Lees (1973) and 2 circadian-based coincidence models proposed by Pittendrigh (1972). Of the circadian models, external coincidence comprises a single oscillator with light having 2 roles, entrainment of the oscillator and coincidence (or not) of light with a particular phase–dubbed the ‘photoinducible phase’ (φ_i_) by Pittendrigh (1966)–thereby discriminating long summer days from the shorter days of approaching autumn. In insects, there is less evidence for an internal coincidence device involving 2, dawn and dusk oscillators, with PPTM being a consequence of changing phase relationship between them as daylength changes with the seasons. External coincidence and hourglass-like models will feature most strongly in this review.

There have been 2 main approaches to the problem of time measurement in PPTM. In the first, the extended ‘nights’ of diapause-inducing, 2 day (say LD 12:36 h, T = 48 h) or 3 day (LD 12:60 h, T = 72 h) cycles are systematically scanned by a short (1 or 2 h) light pulse. This experimental design (Bünsow, 1953) may reveal a light-sensitive phase equivalent to φ_i_ at roughly 24 h intervals in the extended night at which the supplementary light pulse induces nondiapause or a ‘long-day’ effect (Saunders, 1970, 2021), the 24 h intervals between these points acting as evidence for circadian involvement in time measurement, as first suggested by Bünning (1936, 1960). In the second type of experiment, dubbed the Nanda-Hamner (NH) protocol after its instigators (Nanda and Hamner, 1958; see also Teets and Meuti, 2021), the organism under investigation is exposed to a range of abnormal (i.e. non-24 h) LD cycles, each containing the same light phase (say 10 to 12 h in duration) but a variably extended dark phase, to give cycle lengths (T h) ranging up to T = 72 h or more. In insects, the NH protocol has been used more frequently than the Bünsow experiment but has produced variable results. In some species, such as the flesh fly *Sarcophaga argyrostoma* (Saunders, 1973) and its pupal parasitoid *Nasonia vitripennis* (Saunders, 1974) peaks of diapause incidence were observed close to T = 24 h, 48 h, and 72 h, whereas troughs of diapause were close to T = 36 h and 60 h, clearly indicating circadian involvement. The declining amplitude of the diapause peaks with increasing cycle length in *S. argyrostoma* has been attributed to dampening of the circadian oscillation involved (Saunders, 2021). The more complex response in *N. vitripennis*, initially interpreted as evidence for internal coincidence, however, has been re-interpreted as evidence for oscillator dampening in a clock of the external coincidence type (Saunders, 2021)–but this requires further experimental investigation. In some species such as the aphid *Megoura viciae* (Lees, 1986) and drosophilids from high latitudes such as *Drosophila ezoana* (Vaze and Helfrich-Förster, 2016) and *D. montana* (Lankinen et al., 2021) ‘negative’ NH responses, lacking peaks and troughs in diapause incidence, have been recorded. In these cases, PPTM resembles a non-circadian hourglass-like timer. This hourglass-like clock mechanism, however, may be due to a rapidly dampening circadian oscillation, based upon the same molecular components as clocks showing ‘positive’ responses but with extreme reduction of their output (Lewis and Saunders, 1987; Lankinen et al., 2021).

**Figure 9.**
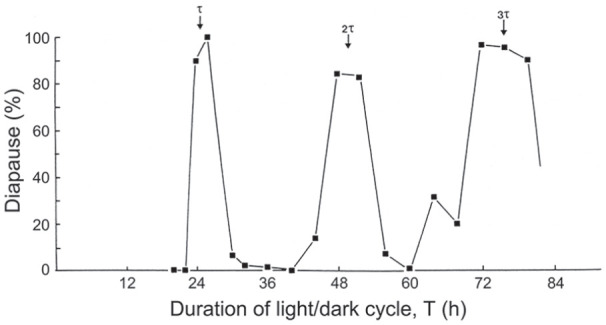
*Calliphora vicina.* Larval diapause induction in Nanda-Hamner (NH) photocycles (see text for details), each consisting of 13 h of light coupled with various hours of darkness to give photocycles (T h) ranging from 18 to 80 h. τ, 2τ and 3τ mark peaks of high diapause incidence occurring at circadian intervals as cycle length is extended. The ‘positive’ NH profile suggests a photoperiodic clock conforming to the ‘external coincidence’ model, with 3 peaks of equal magnitude further suggesting a clock based on an undamped circadian oscillation. From Saunders (1997).

Nanda-Hamner experiments have yet to be attempted in *L. sericata* or *P. terraenovae*, but Figure 9 shows the result of such an experiment using *C. vicina*, plotting the incidence of larval diapause produced by female flies exposed, in different experimental subsets, to LD cycles each containing 13 h of light coupled with an increasing duration of darkness to give photocycles (T h) ranging from 18 to 80 h (Saunders, 1997). The diapause profile shows 3 peaks at approximately 24, 48, and 72 h, or at circadian intervals equivalent to τ, 2τ and 3τ, respectively. Unlike similar published NH results for *S. argyrostoma* (Saunders, 1973) and *N. vitripennis* (Saunders, 1974), the diapause peaks are not reducing in amplitude—if anything, they are becoming more robust as T increases—suggesting that the photoperiodic system underlying the response in *C. vicina* is that of a self-sustained rather than a dampening circadian oscillator.

One of the difficulties in proceeding further with an analysis of such a result is the covert nature of the presumed photoperiodic oscillator. This problem was first recognised by Bünning (1936, 1960) who proposed using an overt rhythm–in his case the up-and-down movements of the leaves of bean seedlings–to act as ‘hands of the (photoperiodic) clock’, assuming that the 2 rhythmic systems had similar properties. In insects, this approach was adopted in an analysis of photoperiodic induction in *S. argyrostoma* (Saunders, 1978) using the rhythm of adult eclosion as an overt indicator of phase. It has also been used for *C. vicina* (Kenny and Saunders, 1991); the results of this study are described below.

Pittendrigh and Minis (1971) observed that the strategy underlying a test of the hypothesis that circadian rhythmicity was involved in PPTM should consist of parallel studies of diapause induction and circadian entrainment preferably in the same species, and that this approach should be based upon phase response curves (PRCs) of the oscillator(s) involved. Systematic studies of PRCs have shown reciprosity between the duration and intensity of light pulses impinging upon an oscillation (Winfree, 1970; Johnson, 1990). In these studies, short duration or low-intensity light pulses have been shown to elicit small phase shifts of an oscillator leading to a ‘Type 1’ PRC with an average slope parallel to light-on, whereas longer and/or higher intensity light pulses generate large phase shifts leading to a ‘Type 0’ PRC with an average slope parallel to light-off (Winfree, 1970). Furthermore, after very long and/or bright photophases (or bright ‘constant’ light) the clock returns to a phase equivalent to that at the beginning of the subjective night, i.e. at a phase denoted as CT 12, the beginning of the subjective night (Pittendrigh, 1966). Figure 3 illustrates this phenomenon in *C. vicina* (Saunders and Cymborowski, 2008). For photoperiodic time measurement in *C. vicina* the consequence of the photoperiodic oscillator being reset to CT 12 at light-off is that the photoinducible phase (φ_i_) predicted by the external coincidence model must lie close to the end of the critical nightlength, i.e. at CT 12 + 9.5 h, or at about CT 21.5 h, late in the night (Saunders, 2021).

**Figure 10.**
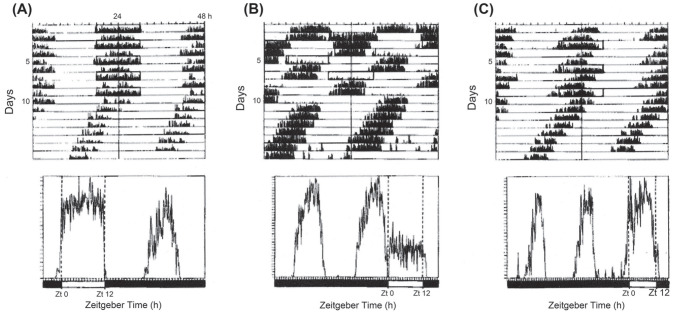
*Calliphora vicina*. The locomotor activity rhythm as overt ‘hands’ of the photoperiodic clock in Nanda-Hamner photocycles. a—LD 12: 36 h (T 48 h); b– LD 12:48 (T 60 h) and C—LD 12: 60 h (T 72 h). In each actograph the 12 h light pulses (open boxes) reset the oscillation to a phase close to Circadian Time 12 (the beginning of the subjective night; see Figure 3). In a and c the activity and the light pulses are in phase, leading to a high incidence of diapause, whereas in b they are out of phase, leading to diapause aversion. Each panel is accompanied by an average activity profile at the relevant T value. From Kenny and Saunders (1991). Abbreviations: LD = light:dark.

The external coincidence model explains the results of Nanda-Hamner experiments with *C. vicina*, as seen in Figure 10 (Kenny and Saunders, 1991). For example, in cycles of LD 12:36 h (T = 48 h) and LD 12:60 h (T = 72 h), which are both multiples of 24 h, the 12 h light components fall at ‘expected’ phases and do not encroach upon the subjective night; hence φ_i_ falls in the dark of each cycle and diapause incidence is high (Figure 10). In LD 12:48 h (T = 60 h), however, the clock is reset each time it is illuminated by the 12 h photophase, causing light to fall in the subjective night and illuminate φ_i_ in some cycles–resulting in nondiapause development (Figure 10).

**Figure 11.**
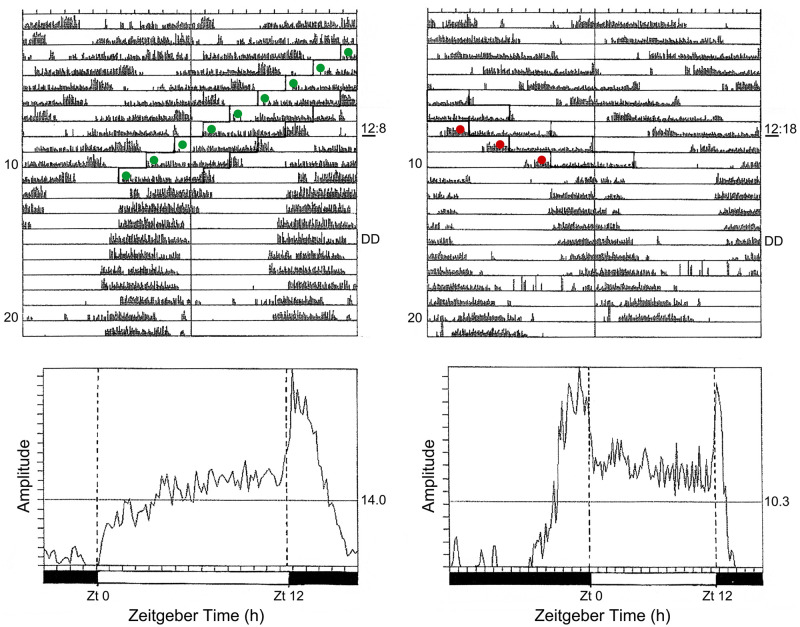
*Calliphora vicina.* Locomotor activity as ‘hands’ of the photoperiodic clock in cycles close to the limits of primary entrainment. *Left*: initial entrainment to LD 12:8 h (T = 20 h), then DD. *Right*: initial entrainment to LD 12:18 h (T = 30 h), then DD. During entrainment to T = 20 h activity phase lags the light and onset of the light pulse encroaches upon the end of the subjective night (and therefore coincides with the photoinducible phase (φ_i_) causing long-day, nondiapause effects. In T = 30 h activity phase leads the light so that the end of the light pulse encroaches upon the early subjective night resulting in a high incidence of diapause. Estimated centroids for φ_i_ falling in the light (nondiapause inducing) are shown as green spots; those falling in the dark (diapause inducing) are in red. Positions of the light pulses (open boxes) are only shown for some of the entrained records. *Lower panels*: average activity profiles during periods of entrainment, showing phase-lagging activity in LD 12:8 h and phase-leading in LD 12:18 h. The peak of activity following light-off in LD 12:18 h is probably due to exogenous masking. From Kenny and Saunders (1991). Abbreviation: DD = constant darkness; LD = light:dark.

Coincidence or non-coincidence of light with φ_i_ is also illustrated in Figure 11 for *C. vicina* exposed to light cycles close to the limits of the oscillator’s primary range of entrainment (Kenny and Saunders, 1991). In a cycle of LD 12:8 h (T = 20 h) some flies entrain to the short period (Figure 11, left-hand panel) with the activity band phase-lagging the light, whereas in flies entraining to LD 12:18 h (T = 30 h) the activity band phase-leads it (Figure 11, right-hand panel). In LD 12:8 h, therefore, light commences during the late subjective night–and must coincide with φ_i_ at CT 21.5 h–whereas in LD 12:18 h the light pulse extends to illuminate phases in the *early* subjective night, leaving φ_i_ in the dark. Entrainment to the short period light cycle thus leads to a low incidence of diapause, whereas that to the longer light cycle results in a high diapause incidence–as seen in the Nanda-Hamner profile for *C. vicina* in Figure 9.

### Output From the Clock: Endocrine and Neural Effectors

Progress has been made in determining details of the regulation of ovarian diapause in *P. terraenovae.* Staining with paraldehyde-fuchsin revealed a cluster of 10 to 14 median neurosecretory cells in each hemisphere of the brain pars intercerebralis (Toyoda et al., 1999). When these cells were surgically removed, the ovaries remained small even under diapause-averting conditions, suggesting that they secrete an allatotropic factor normally stimulating vitellogenesis. A subsequent study using retrograde filling through the cardiac-recurrent nerve with NiCl_2_ (Shiga et al., 2000; Shiga and Numata, 2001) labelled 3 groups of neurons—those in the pars intercerebralis (PI), together with others in the pars lateralis (PL) and in the suboesophageal ganglion—areas possibly involved in conveying the diapause-inducing signals to inhibit release of juvenile hormone from the corpus allatum (CA). Removal of the PI confirmed that the ovaries failed to develop under diapause-averting conditions, whereas when the PL were removed females were prevented from entering diapause. These experiments suggested that neurons in the PI regulate vitellogenesis, whereas those in the PL prevent the onset of diapause. It was later shown that a decrease in the production of juvenile hormone (JHIII bisepoxide) by the corpora allata was the cause of diapause induction, probably in cooperation with unknown allatostatic and allatotropic factors (Shiga et al., 2003).

Much less is known about the control of larval diapause in *C. vicina* which involves a long series of events from the brain-centred photoperiodic clock, through the ovarian egg to the larval progeny, and then through moults of the larvae from first to second instar, and from second to third, before finally entering diapause (or not) in the fully-fed larva. Using an ecdysteroid radioimmunoassay, Richard and Saunders (1987) showed that isolated ring glands (RGs) from *C. vicina* responded to crude extracts from the brains of the flesh fly *Sarcophaga argyrostoma* to produce elevated levels of the moulting hormone ecdysone, but this ability fell to a low basal rate as the larvae entered diapause and could no longer respond to PTTH stimulation, a refractory state that persisted during diapause. If diapausing larvae were subjected to a temperature rise from 11 to 25 °C competency to PTTH stimulation was regained, but no such recovery was possible with isolated brain-RG complexes, indicating a requirement for a ‘whole-body’ temperature rise and a non-cerebral factor.

In *C. vicina*, maternal regulation of larval diapause must mean that eggs laid in the autumn by long-night exposed females are qualitatively different from those deposited during the summer when nights are still short. The ovarian egg is the connection between the generations, but the first two larval-larval moults must then involve the same brain-RG (PTTH-ecdysone) regulation as that in the third instar before the ‘shut down’ of ecdysone production as the larva enters diapause. Almost nothing is known about the nature of this transfer of photoperiodic ‘information’ from the maternal generation to the third instar larva although it may represent an epigenetic phenomenon, perhaps involving DNA methylation regulating gene expression.

## Discussion

The circadian control of locomotor rhythmicity is broadly similar in all of the blow flies considered in this review with regard to persistence in DD, period lengthening in LL and the occurrence of arrhythmicity under continuous bright light. For *C. vicina* and *L. sericata* these aspects of rhythmicity are practically indistinguishable. In *P. terraenovae*, however, arrhythmicity only occurs under much brighter light (up to about 500 lux, Hamasaka et al., 2011), indicating that this species is much less photosensitive.

For photoperiodic induction of diapause the differences between the species are much greater: diapause occurs in the larvae of *C. vicina* and *L. sericata* (Vinogradova and Zinovjeva, 1972; Cragg and Cole, 1952) but as an adult or reproductive diapause in *P. terraenovae* (Vinogradova, 1986; Numata and Shiga, 1995). In *C. vicina*, the external coincidence model suggests how photoperiodic induction in a multivoltine species operates under field conditions. Starting in late summer, while night lengths are still shorter than the critical value, dawn light continues to illuminate the photoinducible phase (φ_i_), thereby maintaining nondiapause development. However, as autumn approaches and night lengths begin to increase beyond the critical value, dawn occurs later and later (with respect to the circadian time scale) until φ_i_ comes to lie in the dark. Long nights are then accumulated by the photoperiodic counter to a threshold at which development is diverted down the diapause pathway. In *C. vicina*, these diapause-inducing effects are then reinforced by continued sensitivity of the resulting larvae to short days and by reduced temperature (Vaz Nunes and Saunders, 1989).

In *C. vicina*, the Nanda-Hamner protocol reveals 3 peaks of high diapause incidence at about 24 h intervals as cycle length is extended (Figure 9) indicating that circadian rhythmicity plays a role in photoperiodic time measurement–specifically the external coincidence model for the clock. Moreover, the 3 high diapause peaks are of equal rather than declining magnitude—unlike the flesh fly *Sarcophaga argyrostoma* (Saunders, 1973)–suggesting that a persistent or undamped circadian oscillator is involved in *C. vicina.*

In *C. vicina* and *L. sericata* the adult and larval stages are both clearly sensitive to the diapause-inducing effects of photoperiod (Vaz Nunes and Saunders, 1989; Tachibana and Numata, 2004a, 2004b). However, a clearly defined CDL has so far only been determined for the maternal generation in *C. vicina;* the external coincidence model, therefore, is currently only clearly applicable to adult females of this species. Larvae of *C. vicina* have yet to be tested for this model.

**Figure 12.**
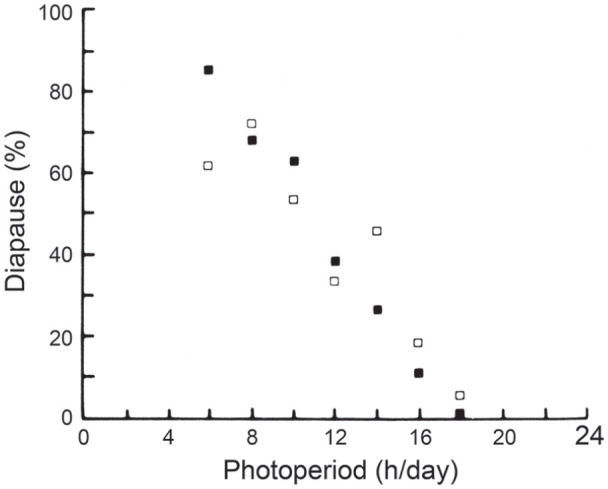
*Lucilia sericata.* Adult flies kept as a mixed-age population under constant light at 25 °C, larvae at range of photoperiods from LD 6: 18 h to LD 18: 6 h showing linear, qualitative relationship between photoperiod and diapause incidence with no evidence of a critical daylength. Open and closed squares show results of replicate experiments. From Saunders et al. (1986). Abbreviations: LD = light:dark.

In *L. sericata*, the maternal generation was exposed to just 2 photoperiods (LD 12:12 and LD 16:8 h), insufficient to establish a CDL (Tachibana and Numata, 2004a). Furthermore, progeny of *L. sericata* produced by a mixed-age population of parental flies kept at 25 °C under continuous light, but then raised as larvae in a range of photoperiods between LD 6: 18 h and LD 18:6 h (Saunders et al., 1986), also showed a linear response, lacking a CDL (Figure 12). Further experiments are clearly needed to see whether a clock of the external coincidence type is generally applicable.

The black blow fly *P. terraenovae* diapauses as an adult. Experiments conducted by Numata and Shiga (1995) have shown a significant larval sensitivity to daylengths between LD 12:12 and 18:6 at 25 °C. Furthermore, although diapause incidence increased as photophase shortened, only 4 photoperiods were studied and the response to them was almost linear, without a well-marked CDL and therefore also lacking clear evidence for external coincidence. A second experiment in which cultures were raised under LD 12:12 or 18:6 h, at temperatures between 17.5 and 30 °C, showed a higher proportion of diapausing adult flies in both photoperiods in the cooler conditions. Short daylength and low temperature were therefore important factors inducing diapause in all flies studied.

*P. terraenovae*, is a northern species occurring in both Subarctic and Arctic regions, most commonly presenting a univoltine life cycle at higher latitudes (Vinogradova, 1986). The prolonged period of winter diapause occurs in the adult fly, the females showing an arrest of follicular development at previtellogenic stages and hypertrophy of the fat body. In diapause, the flies show decreased locomotor activity and a tendency to aggregate in hibernation sites. In the very far north, the early arrival of winter and its delayed departure means that the summer active period is very short and almost entirely occurs in constant light or in very long days. Under these conditions light (photoperiod) must provide an unreliable seasonal signal; consequently low temperature adopts that role (Vinogradova, 1986). At lower latitudes, however, *P. terraenovae* larvae may respond to long daylength and raised temperature to produce nondiapausing flies and the possibility of at least a bivoltine life cycle (Vinogradova, 1986; Numata and Shiga, 1995). Although such larvae are able to discriminate long from short days at 25 °C (Numata and Shiga, 1995) the response to photoperiod is nearly linear between LD 12:12 h and LD 18:6 h.

Photoperiodic response curves showing an abrupt critical value–as in the external coincidence model (e.g. Figure 4 for *C. vicina*)–are generally regarded as examples of *qualitative* time measurement in which day- or nightlength measurement is accomplished by a threshold or an ‘all-or-nothing’ mechanism. In contrast, photoperiodic responses lacking an abrupt critical point, but having a more graded response (e.g. Hardie, 1990; Spieth and Sauer, 1991; Numata and Kobayashi, 1994) are regarded as examples of a *quantitative* response to photoperiod. If this distinction is applied to the photoperiodic responses of the blow flies discussed in this review, the implication might be that different mechanisms of time measurement are employed at different stages of development, even in the same species.

In *C. vicina*, diapause induction in individual insects must show an abrupt, all-or-nothing response directing development along either the diapause or nondiapause pathways, whereas the ‘depth’ or ‘intensity’ of larval diapause–or processes associated with ‘diapause development’ leading to its termination and subsequent post-diapause quiescence (Hodek, 1971)–are probably more continuously expressed. Furthermore, reciprocal crossing experiments between northern and southern strains of *C. vicina* (McWatters and Saunders, 1996) have shown that diapause incidence (i.e. induction) is determined almost entirely by maternal genes, whereas the ‘depth’, or duration, of the resulting diapause is a purely larval phenomenon involving genes from both parents. The difference between qualitative and quantitative photoperiodic responses may therefore be influenced by distinct genetic mechanisms and show a difference in coherence within a group of insects. In responses showing an abrupt critical value–such as that in Figure 4 for *C. vicina*, for example–the larvae were progeny of flies whose circadian rhythmicity was synchronised to CT 12 by transfer from light to darkness and then by laying a single batch of eggs on a particular day post-eclosion; the group, therefore, possessed a high degree of phase coherence with the critical daylength reflecting the qualitative all-or-nothing response at the individual level. It is possible, therefore, that quantitative responses to photoperiod may occur when such coherence between individuals in the population is lacking.

## Future Directions

The short input pathway from photoperiodic reception to adult reproductive diapause in *Protophormia terraenovae* has facilitated significant progress in unravelling the details of photoperiodic induction in that species (Shiga and Numata, 1997, 2001). In *Lucilia sericata* and *Calliphora vicina*, however, larval diapause induced by a maternal sensitivity to photoperiod presents virtually unknown events between photoreception and larval diapause regulation during which photoperiodic ‘information’ is transmitted from the adult female fly to mature post-fed larvae without compromising the normal, intervening, larval moults. This presents a considerable challenge to progress for researchers in insect photoperiodism. Of more immediate interest, however, is the nature of the photoinducible phase: What happens when dawn light coincides with this particular phase during the late subjective night–the crucial event in External Coincidence? One clue may be provided by the results of action spectrum studies in the aphid *Megoura viciae* (Lees, 1981) and the flesh fly *Sarcophaga similis* (Goto and Numata, 2009) which indicate that the early subjective night is maximally sensitive to blue-green light, whereas the late subjective night (the photoinducible phase) shows a much wider peak of sensitivity into longer wavelengths, suggesting that entrainment is effected by cryptochrome, but regulation of the diapause/development switch at the photoinducible phase involves a different photopigment. Characterization of events occurring at this phase–as summer gives way to autumn–is probably the most important outstanding question in insect photoperiodism.

## References

[bibr1-07487304211054419] Amendt J Bugelli V Bernhardt V (2021) Time flies: age grading of adult flies for the estimation of post-mortem interval. Diagnostics 11:152. 33494172 10.3390/diagnostics11020152 PMC7909779

[bibr2-07487304211054419] Aschoff J von Saint Paul U (1982) Circadian rhythms in the blowfly, *Phormia terraenovae*: the period in constant light. Physiol Entomol 7:365-370.

[bibr3-07487304211054419] Bradshaw WE (1976) Geography of photoperiodic response in a diapausing mosquito. Nature 262:384-386. 10.1038/262384b0 8725

[bibr4-07487304211054419] Bünning E (1936) Die endogene Tagesrhythmik als Grundlage der Photoperiodischen Reaktion. Ber Dt Bot Ges 54:590-607.

[bibr5-07487304211054419] Bünning E (1960) Circadian rhythms and time measurement in photoperiodism. Cold Spring Harb Symp Quant Biol 25:249-256. 10.1101/sqb.1960.025.01.029 13711096

[bibr6-07487304211054419] Bünning E Joerrens G (1960) Tagesperiodische antagonistische Schwankungen der Blau-violett und Gelbrot-Empfindlichkeit als Grundlage der photoperiodischen Diapause-Induktion bei Pieris *brassicae*. Z Naturf 15:205-213.

[bibr7-07487304211054419] Bünsow RC (1953) Uber Tages- und Jahresrhythmische Anderungen der Photoperiodischen Lichteropfindlichkeit bei *Kalanchoë blossfeldiana* und ihre Beziehungen zur endogonen Tagesrhythmik. Z Bot 41:257-276.

[bibr8-07487304211054419] Cragg JB Cole P (1952) Diapause in *Lucilia sericata* (Mg.) Diptera. J Exp Biol 29:600-604.

[bibr9-07487304211054419] Coleman PC (2014) The physiology and ecology of diapause under present and future climate conditions in the blow fly, Calliphora vicina [Ph.D. thesis]. Birmingham (UK): University of Birmingham.

[bibr10-07487304211054419] Cymborowski B Hong S-F McWatters HG Saunders DS (1996) S-antigen antibody partially blocks entrainment and the effects of constant light on the rhythm of locomotor activity in the adult blow fly, Calliphora vicina. J Biol Rhythms 11:68-74. 10.1177/074873049601100107 8695894

[bibr11-07487304211054419] Cymborowski B Lewis RD Hong S-F Saunders DS (1994) Circadian locomotor activity rhythms and their entrainment to light-dark cycles continue in flies (*Calliphora vicina*) surgically deprived of their optic lobes. J Insect Physiol 40:501-510.

[bibr12-07487304211054419] Danilevskii AS (1965) Photoperiodism and seasonal development of insects. 1st English ed. Edinburgh and London: Oliver & Boyd Ltd.

[bibr13-07487304211054419] Goto SG (2013) Roles of circadian clock genes in insect photoperiodism. Entomol Sci 16:1-16.

[bibr14-07487304211054419] Goto SG Numata H (2009) Possible involvement of distinct photoreceptors in the photoperiodic induction of diapause in the flesh fly *Sarcophaga similis*. J Insect Physiol 55:401-407. 19084533 10.1016/j.jinsphys.2008.11.008

[bibr15-07487304211054419] Hall M Wall R (1995) Myiasis of humans and domestic animals. Adv Parasitol 35:257-334. 7709854 10.1016/s0065-308x(08)60073-1

[bibr16-07487304211054419] Hamasaka Y Watari Y Arai T Numata H Shiga S (2001) Retinal and extraretinal pathways for entrainment of the circadian activity rhythm in the blow fly, *Protophormia terraenovae*. J Insect Physiol 47:867-875.

[bibr17-07487304211054419] Hamasaka Y Watari Y Arai T Numata H Shiga S (2011) Comparison of the effect of constant light on the circadian rhythm of white-eye and wild-type blow fly *Protophormia terraenovae*. Biol Rhythm Res 42:303-311.

[bibr18-07487304211054419] Hardie J (1990) The photoperiodic counter, quantitative day-length effects and scotophase timing in the vetch aphid *Megoura viciae*. J Insect Physiol 36:939-949.

[bibr19-07487304211054419] Harvey ML Dadour IR Gasz NE (2021) Maggot therapy in chronic wounds: new approaches to historical practices. Ann Ent Soc Am 114:415-424.

[bibr20-07487304211054419] Helfrich C Cymborowski B Engelmann W (1985) Circadian activity rhythm of the house fly continues after optic tract severance and lobectomy. Chronobiol Intern 2:19-32. 10.3109/07420528509055538 3870837

[bibr21-07487304211054419] Helfrich-Förster C Winter C Hofbauer A Hall JC Stanewsky R (2001) The circadian clock of fruit flies is blind after elimination of all known photoreceptors. Neuron 30:249-261. 11343659 10.1016/s0896-6273(01)00277-x

[bibr22-07487304211054419] Hodek I (1971) Termination of adult diapause in *Pyrrhocoris apterus* (Heteroptera: Pyrrhocoridae) in the field. Entomol Exp Appl 14:212-222.

[bibr23-07487304211054419] Hong S-F Saunders DS (1994) Effects of constant light on the rhythm of adult locomotor activity in the blowfly, *Calliphora vicina*. Physiol Entomol 19:319-324.

[bibr24-07487304211054419] Johnson CH (1990) Forty years of PRCs: what have we learned? Chronobiol Internat 16:711-743. 10.3109/07420529909016940 10584173

[bibr25-07487304211054419] Kasai M Chiba Y (1987) Effect of optic lobe ablation on circadian activity in the mosquito, *Culex pipiens pallens*. Physiol Entomol 12:59-65.

[bibr26-07487304211054419] Kenny NAP Saunders DS (1991) Adult locomotor rhythmicity as ‘hands’ of the maternal photoperiodic clock regulating larval diapause in the blowfly, *Calliphora vicina*. J Biol Rhythms 6:217-233. 1773093 10.1177/074873049100600303

[bibr27-07487304211054419] Lankinen P (1986) Geographical variation in circadian eclosion rhythm and photoperiodic adult diapause in *Drosophila littoralis*. J Comp Physiol A 159:123-142. 10.1177/074873048600100202 2979577

[bibr28-07487304211054419] Lankinen P Kastally C Hoikkala A (2021) Nanda-Hamner curves show huge latitudinal variation but no circadian components in *Drosophila montana* photoperiodism. J Biol Rhythms 20:1-13. 10.1177/0748730421997265 PMC8114436 33745359

[bibr29-07487304211054419] Lees AD (1973) Photoperiodic time measurement in the aphid *Megoura viciae*. J Insect Physiol 19:2279-2316.

[bibr30-07487304211054419] Lees AD (1981) Action spectra for the photoperiodic control of polymorphism in the aphid *Megoura viciae*. J Insect Physiol 27:761-771.

[bibr31-07487304211054419] Lees AD (1986) Some effects of temperature on the hourglass photoperiod timer in the aphid *Megoura viciae*. J Insect Physiol 32:79-89.

[bibr32-07487304211054419] Lewis RD Saunders DS (1987) A damped circadian oscillator model of an insect photoperiodic clock. I. Description of the model based on a feedback control system. J Theoret Biol 128:47-59.

[bibr33-07487304211054419] McWatters HG Saunders DS (1996) The influence of each parent and geographic origin on larval diapause in the blow fly, *Calliphora vicina*. J Insect Physiol 42:721-726. 10.1016/s0022-1910(97)00051-6 12770449

[bibr34-07487304211054419] McWatters HG Saunders DS (1997) Inheritance of the photoperiodic response controlling larval diapause in the blow fly, *Calliphora vicina*. J Insect Physiol 43:709-717. 12770449 10.1016/s0022-1910(97)00051-6

[bibr35-07487304211054419] McWatters HG Saunders DS (1998) Maternal temperature has different effects on the photoperiodic response and duration of larval diapause in blow fly (*Calliphora vicina*) strains collected at two latitudes. Physiol Entomol 23:369-375.

[bibr36-07487304211054419] Nanda KK Hamner KC (1958) Studies on the nature of the endogenous rhythm affecting photoperiodic response of Biloxi soybean. Bot Gaz 120:14-25.

[bibr37-07487304211054419] Numata H Kobayashi S (1994) Threshold and quantitative responses exist in an insect. Experientia 50:969-971.

[bibr38-07487304211054419] Numata H Shiga S (1995) Induction of adult diapause by photoperiod and temperature in *Protophormia terraenovae* (Diptera: Calliphoridae). Environ Entomol 24:1633-1636.

[bibr39-07487304211054419] Pittendrigh CS (1966) The circadian oscillation in *Drosophila pseudoobscura* pupae: a model for the photoperiodic clock. Z Pflanzenphysiol 54:275-307.

[bibr40-07487304211054419] Pittendrigh CS (1972) Circadian surfaces and the diversity of possible roles of circadian organization in photoperiodic induction. Proc Natl Acad Sci USA 69:2734-2737. 4506793 10.1073/pnas.69.9.2734 PMC427028

[bibr41-07487304211054419] Pittendrigh CS Minis DH (1971) The photoperiodic time measurement in *Pectinophora gossypiella* and its relation to the circadian system in that species. In: Menaker M , editor. Biochronometry. Washington (DC): National Academy of Sciences. p. 212-250. 10.1073/pnas.66.3.758 PMC283115 5269238

[bibr42-07487304211054419] Richard DS Saunders DS (1987) Prothoracic gland function in diapause and non-diapause *Sarcophaga argyrostoma* and *Calliphora vicina*. J Insect Physiol 33:385-392.

[bibr43-07487304211054419] Saunders DS (1966) Larval diapause of maternal origin. II. The effect of photoperiod and temperature on *Nasonia vitripennis*. J Insect Physiol 12:569-581.

[bibr44-07487304211054419] Saunders DS (1970) Circadian clock in insect photoperiodism. Science 169:601-603. 17806783 10.1126/science.168.3931.601

[bibr45-07487304211054419] Saunders DS (1971) The temperature-compensated photoperiodic clock ‘programming’ development and pupal diapause in the flesh-fly *Sarcophaga argyrostoma*. J Insect Physiol 17:801-812.

[bibr46-07487304211054419] Saunders DS (1973) The photoperiodic clock in the flesh fly, *Sarcophaga argyrostoma*. J Insect Physiol 19:1941-1954. 4752418 10.1016/0022-1910(73)90188-1

[bibr47-07487304211054419] Saunders DS (1974) Evidence for ‘dawn’ and ‘dusk’ oscillators in the *Nasonia* photoperiodic clock. J Insect Physiol 20:77-88.

[bibr48-07487304211054419] Saunders DS (1978) An experimental and theoretical analysis of photoperiodic induction in the flesh-fly, *Sarcophaga argyrostoma*. J Comp Physiol A 124:75-95.

[bibr49-07487304211054419] Saunders DS (1981) Insect photoperiodism: the clock and the counter. Physiol Entomol 6:99-116.

[bibr50-07487304211054419] Saunders DS (1987) Maternal influence on the incidence and duration of larval diapause in *Calliphora vicina*. Physiol Entomol 12:331-338.

[bibr51-07487304211054419] Saunders DS (1997) Insect circadian rhythms and photoperiodism. Invert Neurosci 3:155-164. 9783440 10.1007/BF02480370

[bibr52-07487304211054419] Saunders DS (2001) Geographical strains and selection for the diapause trait in *Calliphora vicina*. In: Denlinger DL Giebultowicz JM Saunders DS editors. Insect Timing: Circadian Rhythmicity to Seasonality. Amsterdam: Elsevier. p. 113-121.

[bibr53-07487304211054419] Saunders DS (2020) Dormancy, diapause, and the role of the circadian system in insect photoperiodism. Annu Rev Entomol 65:1-17. 31594413 10.1146/annurev-ento-011019-025116

[bibr54-07487304211054419] Saunders DS (2021) Insect photoperiodism: Bünning’s hypothesis, the history and development of an idea. Eur J Entomol 118:1-13.

[bibr55-07487304211054419] Saunders DS Cymborowski B (1996) Removal of optic lobes of adult blow flies (*Calliphora vicina*) leaves photoperiodic induction of larval diapause intact. J Insect Physiol 42:807-811.

[bibr56-07487304211054419] Saunders DS Cymborowski B (2008) Light induced behavioural effects on the locomotory activity rhythm of the blow fly, *Calliphora vicina*. Eur J Entomol 105:585-590.

[bibr57-07487304211054419] Saunders DS Hayward SAL (1998) Geographical and diapause-related cold tolerance in the blow fly, *Calliphora vicina*. J Insect Physiol 44:541-551. 12769936 10.1016/s0022-1910(98)00049-3

[bibr58-07487304211054419] Saunders DS Hong S-F (2000) Effects of temperature and temperature-steps on circadian locomotor rhythmicity in the blow fly Calliphora vicina. J Insect Physiol 46:289-295. 12770234 10.1016/s0022-1910(99)00182-1

[bibr59-07487304211054419] Saunders DS Macpherson JN Cairncross KD (1986) Maternal and larval effects of photoperiod on the induction of larval diapause in two species of fly, *Calliphora vicina* and *Lucilia sericata*. Exp Biol 46:51-58. 3817113

[bibr60-07487304211054419] Shiga S Numata H (1997) Induction of reproductive diapause via perception of photoperiod through the compound eyes in the adult blow fly, *Protophormia terraenovae*. J Comp Physiol A 181:35-40.

[bibr61-07487304211054419] Shiga S Numata H (2001) Anatomy and functions of the brain neurosecretory neurons with regard to reproductive diapause in the blow fly *Protophormia terraenovae*. In: Denlinger DL Giebultowicz J Saunders DS , editors. Insect Timing: Circadian Rhythmicity to Seasonality. Amsterdam: Elsevier. p. 69-83.

[bibr62-07487304211054419] Shiga S Hamanaka Y Tatsu Y Okuda T Numata H (2003) Juvenile hormone biosynthesis in diapause and nondiapause females of the adult blow fly *Protophormia terraenovae*. Zool Sci 20:119-120. 10.2108/zsj.20.1199 14569142

[bibr63-07487304211054419] Shiga S Toyoda I Numata H (2000) Neurons projecting to the retrocerebral complex of the adult blow fly *Protophormia terraenovae*. Cell Tissue Res 299:427-439. 10772257 10.1007/s004419900110

[bibr64-07487304211054419] Smith PH (1983) Circadian control of spontaneous flight activity in the blowfly, *Lucilia cuprina*. Physiol Entomol 8:73-82.

[bibr65-07487304211054419] Spieth HR Sauer KP (1991) Quantitative measurement of photoperiods and its significance for the induction of diapause in *Pieris brassicae* (Lepidoptera, Pieridae). J Insect Physiol 37:231-238.

[bibr66-07487304211054419] Tachibana S-I Numata H (2004a) Maternal induction of larval diapause and its sensitive stage in the blow fly *Lucilia sericata* (Meigen) (Diptera: Calliphoridae). Entomol Sci 7:231-235.

[bibr67-07487304211054419] Tachibana S-I Numata H (2004b) Parental and direct effects of photoperiod and temperature on the induction of larval diapause in the blow fly *Lucilia sericata*. Physiol Entomol 29:39-44.

[bibr68-07487304211054419] Teets NM Meuti ME (2021) Hello darkness, my old friend: a tutorial of Nanda-Hamner protocols. J Biol Rhythms 20:1-5. 10.1177/0748730421998469 33715479

[bibr69-07487304211054419] Toyoda I Numata H Shiga S (1999) Role of the median neurosecretory cells in the ovarian development of the blow fly *Protophormia terraenovae*. Zool Sci 16:187-191.

[bibr70-07487304211054419] Vaze KM Helfrich-Förster C (2016) *Drosophila ezoana* uses an hour-glass or highly damped circadian clock for measuring night length and inducing diapause. Physiol Entomol 41:378-389. 27867253 10.1111/phen.12165 PMC5108423

[bibr71-07487304211054419] Vaz Nunes M Saunders DS (1989) The effect of larval temperature and photoperiod on the incidence of larval diapause in the blowfly, *Calliphora vicina*. Physiol Entomol 14:471-474.

[bibr72-07487304211054419] Vaz Nunes M Saunders DS (1999) Photoperiodic time measurement in insects: a review of clock models. J Biol Rhythms 14:84-104. 10194645 10.1177/074873049901400202

[bibr73-07487304211054419] Vaz Nunes M Kenny NAP Saunders DS (1990) The photoperiodic clock in the blowfly *Calliphora vicina*. J Insect Physiol 36:61-67.

[bibr74-07487304211054419] Vinogradova EB (1974) The pattern of reactivation of diapausing larvae of the blowfly, Calliphora vicina. J Insect Physiol 20:2487-2496. 4436591 10.1016/0022-1910(74)90033-x

[bibr75-07487304211054419] Vinogradova EB (1986) Geographical variation and ecological control of diapause in flies. In: Taylor F Karban R , editors. The Evolution of Insect Life Cycles. New York: Springer-Verlag. p. 35-47.

[bibr76-07487304211054419] Vinogradova EB (1991) Diapause of flies and its regulation. Trudy Zool Inst Akad Nauk SSSR 118:1-254. (In Russian)

[bibr77-07487304211054419] Vinogradova EB Zinovjeva KB (1972) Maternal induction of larval diapause in the blowfly, *Calliphora vicina*. J Insect Physiol 18:2401-2409. 4641412 10.1016/0022-1910(72)90184-9

[bibr78-07487304211054419] Warman GR Lewis RD (2001) Molecular simulation modelling of the circadian system of the blow fly, *Lucilia cuprina*. J Insect Physiol 47:923-934.

[bibr79-07487304211054419] Winfree AT (1970) Integrated view of resetting a circadian clock. J Theoret Biol 28:327-374. 5487648 10.1016/0022-5193(70)90075-5

